# Infrared exposure improves human colour vision undermined by LED light

**DOI:** 10.1371/journal.pone.0345340

**Published:** 2026-09-25

**Authors:** Mark Robinson, Edward Barrett, Glen Jeffery

**Affiliations:** University College London, Institute of Ophthalmology and Bartlett School of Architecture, United Kingdom of Great Britain and Northern Ireland; Save Sight Institute, AUSTRALIA

## Abstract

Life evolved under sunlight (200–4000nm). Incandescent lighting was similar (200–3000nm). But modern light-emitting diodes (LED) produce only a narrow band of visible light (400–700nm), eliminating >90% of the solar spectrum. Light impacts life, partly through mitochondrial modulation with different wavelengths triggering opposing metabolic responses. Longer red/infrared wavelengths (650–1000nm) improve metabolism and ageing patterns, while blue light (420–450nm) undermines these processes. The retina has the highest metabolic rate in the body and the greatest density of mitochondria. Hence, visual ability may be vulnerable to changes in the light environment which negatively affects mitochondrial performance. We ask if standard LED lighting reduces visual performance in terms of colour discrimination ability, and whether adding 850nm infrared improves it. We tested colour discrimination with a standard Farnsworth-Munsell 100-Hue test in 55 subjects then divided them into those exposed to standard LED desk lighting or LED lights supplemented with 850nm infrared for a week. Controls were unexposed. There were no changes in controls but significant reductions of 33% in colour performance scores in the LED group and significant improvements of 23% in colour performance scores in those supplemented with 850nm. The implications for public health warrant further investigation as LED lighting continues to be widely adopted in the built environment.

## Introduction

Life evolved under full spectrum sunlight for billions of years (200–4000nm) [1]. Fire light and incandescent lighting were similar in spectral range as both are black body sources (200–3000nm) [2]. But modern light-emitting diodes (LED) that now dominate the built environment produce only a narrow band of visible light (400–700nm). This eliminates >90% of the solar spectrum, restricting light to only the human visual sensitive range. LEDs have been adopted for economic reasons that consider the production of infrared (700–1000nm) as wasteful because it is beyond the human visual range (400–700nm). Further, we now spend >90% of our time in the built environment under LED lighting [3], hence they are now a dominant feature of modern human life.

Light impacts life, partly through mitochondrial modulation with different wavelengths triggering opposing metabolic responses in a highly conserved manner from insect to humans. Longer red/infrared wavelengths (650–1000nm) are associated with improved mitochondrial function including increased membrane potential, improved ATP production along with reductions in inflammatory markers [review 4–6 *in vivo*], all of which are key factors in ageing [7]. In insects, improved mitochondrial performance significantly increases life span and aged mobility *in vivo* [8]. Doses, schedules and study types for the cited literature are summarised in Table 1. Improved mitochondrial production via longer wavelength exposure also reduces blood sugars in insects and humans because this results in increased mitochondrial demand for sugars along with oxygen to produce higher levels of ATP *in vivo* [9–10. See Table 1]. Shorter wavelengths, specifically 420–450nm are absorbed by mitochondria probably via their porphyrin [11]. The results are elevated blood sugars [12], reduced mitochondrial membrane potential and ATP production and shorter insect lifespan *in vivo* [10, 13, 14. See Table 1].

**Table 1:** Methodological summary of cited light exposure studies.

Ref	Study	Animal Type	Wavelength used (nm)	Irradiance (source units; mW/cm², brackets where derived)	Acute/ chronic study
[5]	Begum 2013	Animal (mouse)	670	20 mW/cm² at LED face; 0.85 W/m² at cage floor [= 0.085 mW/cm² delivered, derived]	Acute-rep
[6]	Gkotsi 2014	Animal (mouse)	670	40 mW/cm²	Acute-rep
[8]	Begum 2015	Animal (*Drosophila*)	670	40 mW/cm²	Chronic
[9]	Powner & Jeffery 2024	Human	670	40 mW/cm² to 800 cm² of upper back	Acute
[10]	Powner & Jeffery 2022	Animal (bumblebee)	670 + 420	40 mW/cm² (both wavelengths)	Acute
[11]	Kaynezhad 2022	Animal (mouse)	420	~5 W/m² at corneal surface [= ~ 0.5 mW/cm² derived]	Acute
[12]	Cheung 2016	Human	468	Not reported	Acute
[13]	Hoh Kam 2021	Animal (*Drosophila*)	420	40 mW/cm²	Acute-rep + acute
[14]	Nash 2019	Animal (*Drosophila*)	~460	Not reported	Chronic
[16]	Barrett & Jeffery 2026	Human (field)	Broad-spectrum (inc.)	3.7 W/m² on working plane [= 0.37 mW/cm² derived]	Chronic
[17]	Jeffery 2025	Human	850	9 mW/cm² at bare upper back (panel spec 9.18 mW/cm² at 50 cm)	Acute
[22]	Kaynezhad 2016	Animal (rat)	670	Not reported	Acute
[23]	Shinhmar 2021	Human	670	~8 mW/cm² at cornea	Acute
[24]	Amaroli 2024	*In vitro* (mitochondria)	810	0.25, 0.5, 1, 2 W on 1 cm² spot [= 250, 500, 1000, 2000 mW/cm² derived as power/area]	Acute
[26]	Gopalakrishnan 2020	Animal (rat)	830	25 mW/cm² at corneal surface	Chronic
[28]	Françon 2024	*In vitro* (RPE cells)	White-LED, 455, 630	Not reported	Acute
[29]	Núñez-Álvarez & Osborne 2019	*In vitro* + animal (rat)	470 + 630	*In vitro*: blue 12.08, red 6.5 W/m². *In vivo* at cornea: blue 26.3, red 16.5 W/m² [= 0.65–2.63 mW/cm²]	Acute
[30]	Heinig 2020	*Ex vivo* (mouse eyeball)	405 + 670 + 810	1 mW/cm² (blue 405); 60 mW/cm² (red 670 and NIR 810)	Acute
[34]	Al-Hussaini 2025	Animal (mouse)	420 + 450	~13 mW/cm² at cage floor (both wavelengths)	Chronic

*Table 1 Legend. Studies using light on varied subjects, their wavelength and irradiance and whether the study was acute or chronic. Abbreviations: inc. = incandescent; RPE = retinal pigment epithelium; nm = nanometre; mW/cm^2^ = milliwatts per square centimetre; Not reported = mW/cm^2^ not specifically reported in the source paper. Units in square brackets have been converted to mW/cm^2^ where other irradiance metrics have been given. Acute = single exposure; “-rep” = multiple exposures; “Chronic” = sustained or continuous exposure.*

During evolution these two wavelength ranges were in balance under sunlight. This has changed radically in the last 20 years with the introduction of LED lighting where the infrared has been removed and the spectral balance significantly shifted to shorter wavelengths that can undermine mitochondria.

The retina has the greatest mitochondrial content in the body and the highest metabolic demand [15]. It is also uniquely exposed to environmental light because of its clear optics. Recent work has shown that supplementing LED lighting with wide-spectrum incandescent luminaires significantly improves long-term colour vision [16. See Table 1], establishing that spectral modification of the built environment can benefit visual health. However, incandescent supplementation carries potential increased energy consumption that may restrict its general adoption. Hence, we ask if exposure to standard LED lighting reduces colour vision performance, and whether supplementing these with 850nm infrared wavelengths known to penetrate the human body [17] can achieve similar benefits in visual performance in terms of colour discrimination ability. We use standard white LED desk lamps in open office environments with ceiling-mounted fluorescent strips as background lighting and test colour vision with a Farnsworth-Munsell 100-Hue standard colour discrimination test [18].

## Materials and methods

Fifty-five healthy adults (age 22–69 years; mean 40.3 ± 11.8 years; 23 Male, 32 Female) were enrolled in a parallel-group, single-blind, randomised controlled trial and allocated to LED (n = 15; Male:Female ratio 7:8; mean age 38.0 ± 12.6 years), LED + 850 (n = 20; Male:Female 10:10; mean age 41.0 ± 13.1 years), or control (n = 20; Male:Female 6:14; mean age 41.3 ± 10.1 years) groups. Participants were randomised to group at the point of recruitment using random number tables for allocation, capping intervention group LED + 850 at n = 20. Inclusion criteria were healthy adults in ≥20 hours per week of indoor desk-based work. Exclusion criteria were self-reported ocular pathology, intraocular lens implants, neurological conditions, and systemic health conditions. Subjects had best corrected vision including the use of glasses. The study was carried out at University College London. Participants were recruited from within the UCL environment by public notice. The study was approved by University College London Research Ethics Committee (ref 1286/28.07.2025). All participants were fully informed and provided written consent. The project ran from 01/09/2025–05/12/2025.

The LED device was a 15W R63 warm white LED lamp emitting 400–700nm. The LED + 850 device was identical but supplemented with an Epileds diode emitting ~840–870nm infrared, peak 850nm (Deep Red R63 Lamp), both devices from Light Power Health UK. Control participants were exposed to standard fluorescent office ceiling lighting. LED devices were visually indistinguishable; participants were blinded to allocation. Recruitment and allocation were carried out by one author (G.J.) and assessment by another (M.R.). Assessors were not blinded, consistent with a single-blind design.

**Fig 1:**
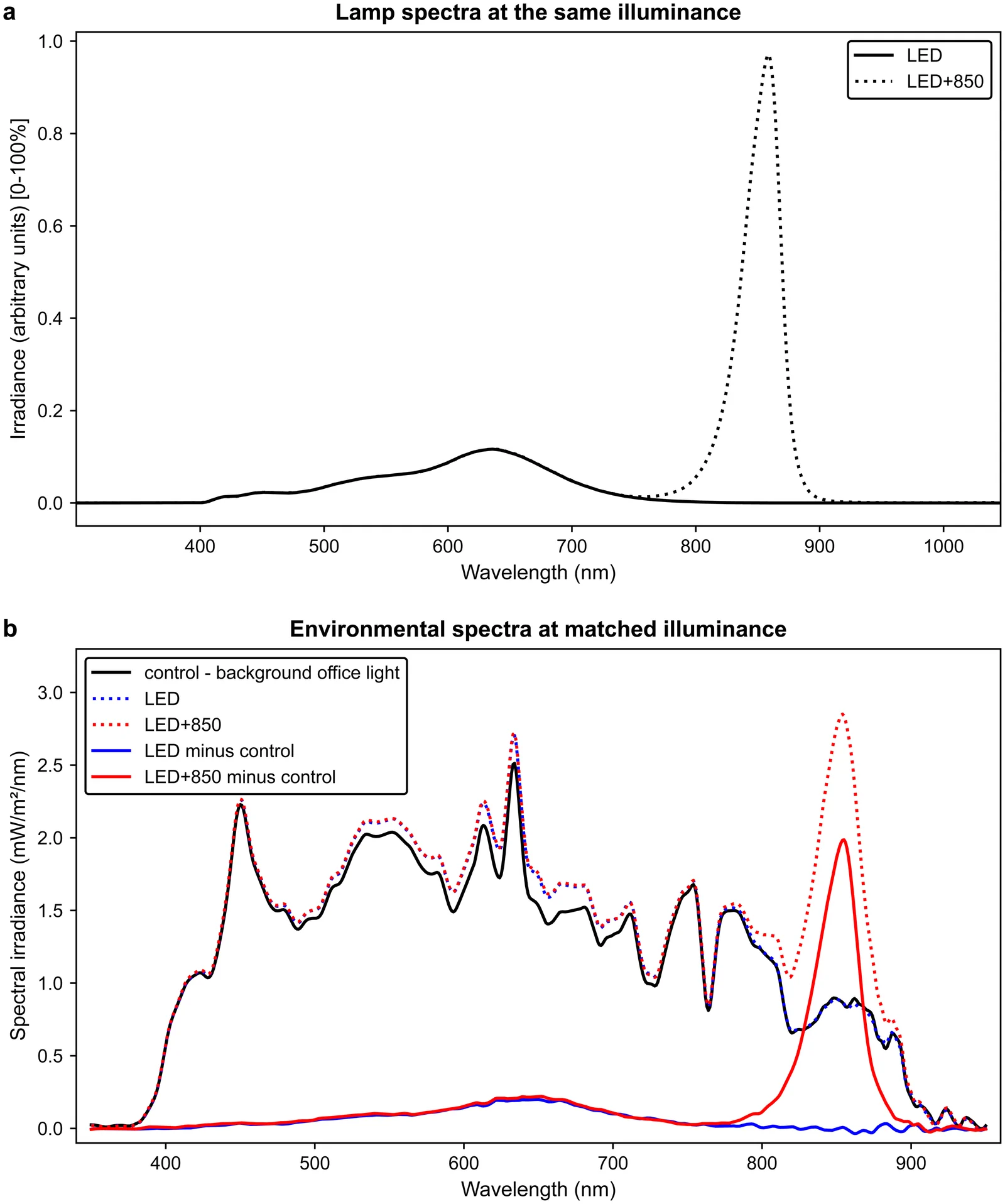
a. Spectral distribution of light sources used in the study. LED light (LED) and infrared-supplemented LED (LED + 850). LED + 850 has identical visible spectrum to LED with added invisible infrared peak at 850nm. Participants could not distinguish the difference between the two LED devices. LED luminaires were mounted at <1m from head height oriented downward, to be comfortable and within the normal range of office light exposure. Spectral irradiance measurements were made using a spectrophotometer (Ocean Optics SR-6XR250-50) with a cosine-corrected optic fibre. For the sake of comparison in panel a, the magnitude of each irradiance spectrum was then adjusted to give the same illuminance value across all spectra. It can be seen from the area under the curves that the LED that included the 850nm will consume more energy to deliver the same illuminance as the LED only lamp. **b.** Spectral irradiance of the light sources in experimental conditions, measured in a typical office. The control is existing office light with the devices off. The LED and LED + 850 are each lamp on in addition to existing office light. The two devices are set to the same illuminance. LED minus control and LED + 850 minus control isolate the light each lamp adds. The LED + 850 minus control shows the 850 nm infrared peak added by the LED + 850 lamp, invisible to the participants.

Participants in LED and LED + 850 groups positioned desk lamps within one meter of their workspace. Lamp placement was directed downward to provide diffuse reflected illumination. All groups maintained ≥20 hours per week exposure for seven days in office and home environments. The spectral distribution of the different forms of lighting is shown in Fig 1a.

Light was also measured in the participants’ working environments. Horizontal illuminance at head height was in the range of 250–1400 lux. Irradiance ranged from 0.7 to 5.0 W/m^2^ (0.07 to 0.50 mW/cm^2^). In a typical office, both the LED and LED + 850 lamps delivered a vertical illuminance at eye of approximately 8 lux. At this matched illuminance the infrared component of the LED + 850 lamp delivered 0.11 W/m^2^ (0.011 mW/cm^2^), nearly four times the 0.03 W/m^2^ (0.003 mW/cm^2^) of the LED lamp alone, the difference being the added 850nm infrared (Fig 1b).

**Fig 2:**
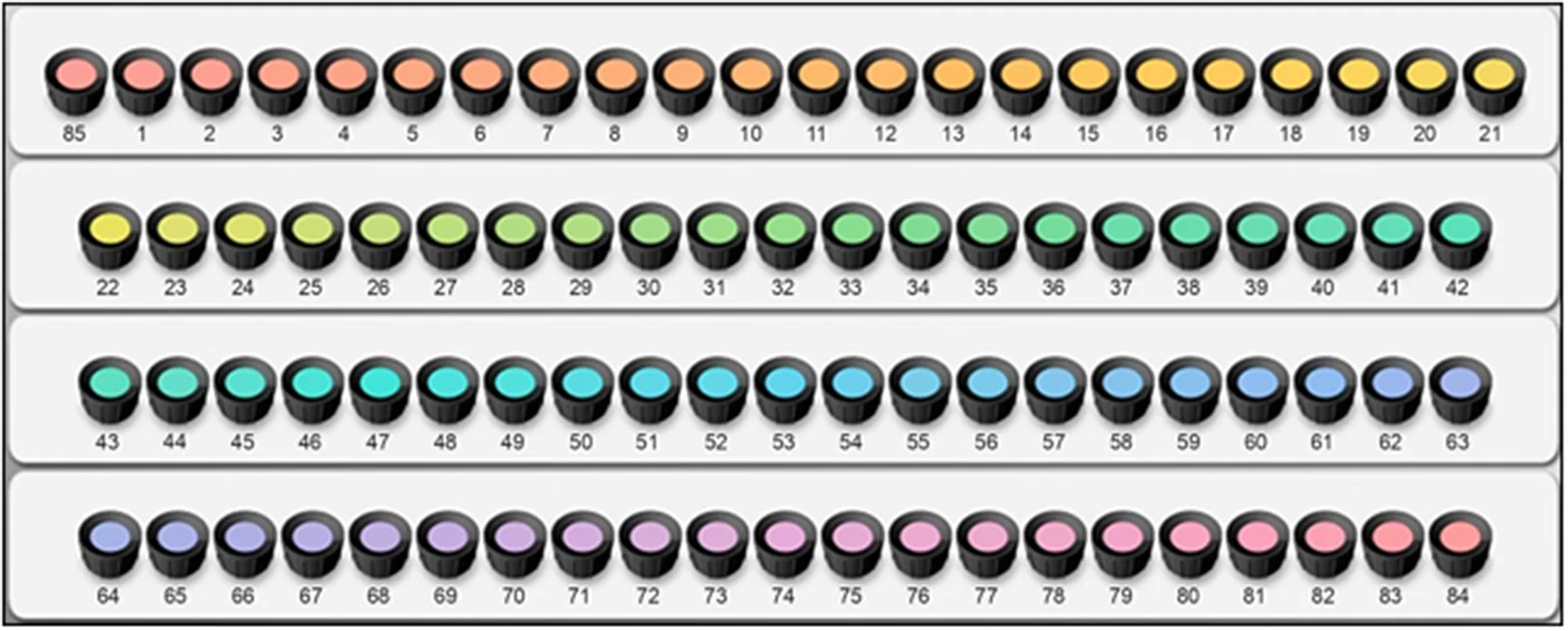
Farnsworth-Munsell 100-Hue test kit. This is a standard sensitive measure of colour discrimination where participants arrange 85 coloured caps in order of hue. Error scores quantify colour discrimination ability. Lower scores indicate superior performance. Participants generally completed the test in approximately 8-10 min. There was no time constraint.

Binocular colour vision was assessed using the Farnsworth-Munsell 100-Hue test (Fig 2) [18] at two timepoints: at baseline on day 1 (before first exposure) and on day 8 (the day after the final exposure). Testing used a standardised LED View Ultimate viewing cabinet under TL84 illumination, in a dimly illuminated environment. Total Error (TE) scores were calculated. Lower scores indicate better performance. Vingrys and King-Smith [19] indices were also calculated from each cap arrangement: the confusion index (*C*, scaled so a perfect arrangement scores 1.0), the selectivity index (*S*), and the confusion angle (*θ*).

**Statistical analysis**: Change scores were tested for normality with the Shapiro-Wilk test. One-way ANOVA compared change in TE scores between groups, and *post hoc* independent samples t-tests for pairwise comparisons corrected for multiple comparisons using Fisher’s protected least significant difference [20]. ANOVA is robust to the single non-normal group, the groups had equal variances (Levene’s test, *p* = 0.535), and the non-parametric Kruskal-Wallis test gave the same result (*p* < 0.001). Within-group changes were assessed using Wilcoxon signed-rank tests because the LED change scores were not normally distributed. Significance threshold was set at p < 0.05. There were no missing data.

## Results

**Fig 3:**
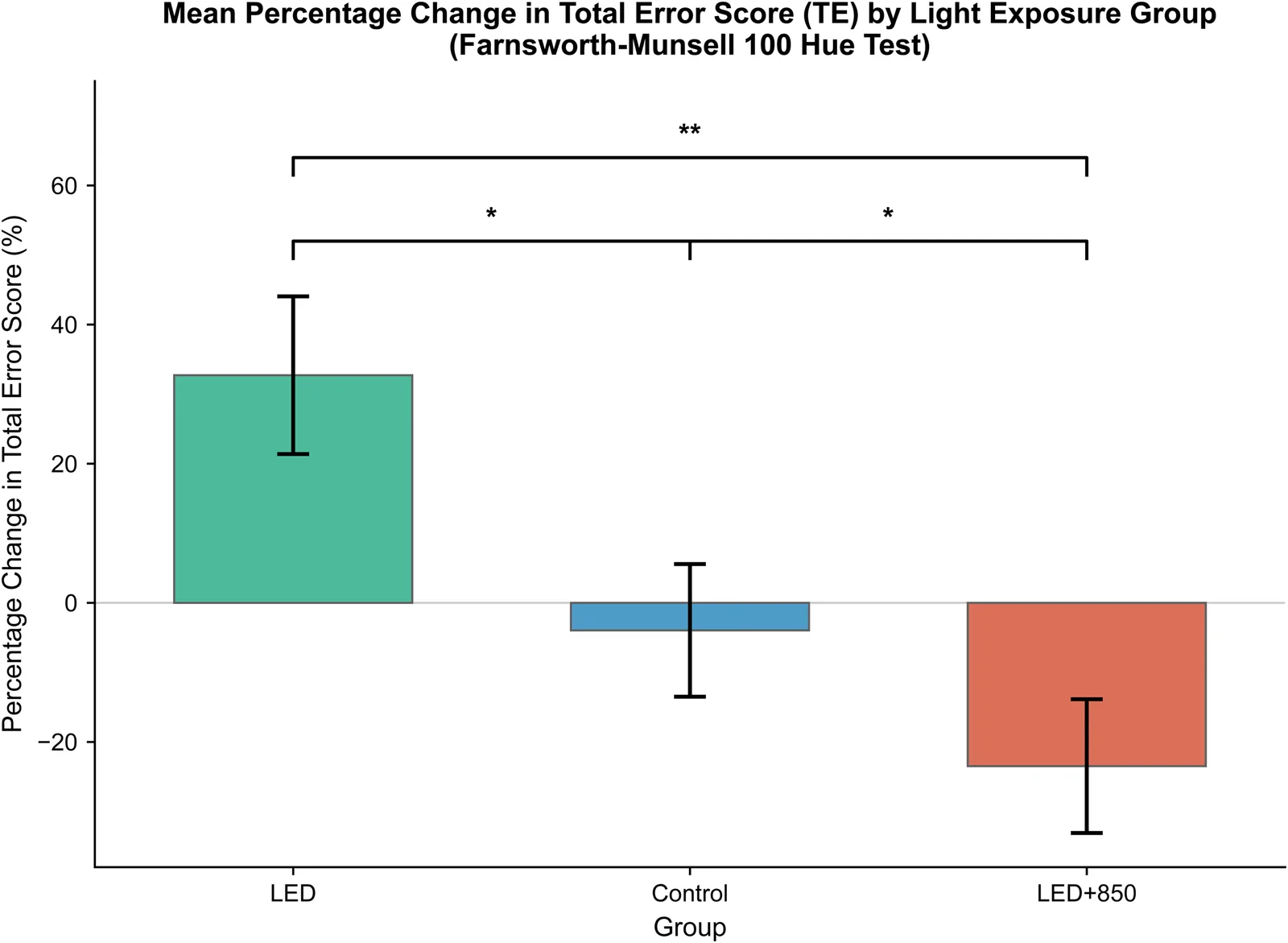
Plot of mean percentage change in Total Error (TE) score by group after 7 days exposure to the different light sources using the Farnsworth-Munsell 100-Hue Test. LED lighting resulted in a significant increase in error scores (Green Bar, left), while the addition of 850nm to this lamp resulted in a significant decline in error scores (Red Bar, right). There were no changes in the control group, (Blue Bar, centre). Error bars denote standard error; negative values indicate improvement (lower TE). Effects of LED light (LED) and Infrared-supplemented LED (LED + 850) on colour vision. Abbreviations: *p < 0.05, **p < 0.01. Group sizes were n = 15 (LED), n = 20 (LED + 850) and n = 20 (control). Significance was tested by one-way ANOVA with independent-samples t-test post-hoc, corrected for multiple comparisons using Fisher’s protected least significant difference.

Fig 3 shows the outcome of the testing which was undertaken 24h after the participants’ last exposure to the two different forms of LED lighting. Participants exposed to LED light (n = 15) had a significant increase in error scores of 33%, revealing a decline in colour discrimination in before and after measurement (p < 0.05). This difference was also statistically significant from controls who were not exposed to either luminaire (p < 0.05). Subjects exposed to the same LED with added 850nm LED (n = 20) had significantly reduced error scores in a before and after measurement of 23% (p < 0.05) consistent with the addition improving colour discrimination. This difference was also significant against controls (p < 0.05) and against subjects exposed to the standard LED without the 850nm addition (p < 0.01). There was no significant change in the control subjects who were not exposed to either luminaire over the test period (n = 20). One-way ANOVA revealed a significant effect of lighting condition on change in TE scores (F(2,52) = 8.26, *p* < 0.001, η² = 0.24). Between-group differences in mean change score were −19.3 TE points (95% CI [−29.8, −8.9]; *d* = −1.29) for LED + 850 versus LED, −9.9 points (95% CI [−18.6, −1.2]; *d* = −0.73) for LED + 850 versus control, and +9.4 points (95% CI [0.1, 18.8]; *d* = 0.70) for LED versus control; negative differences indicate greater improvement. All three protected pairwise comparisons were significant. The confusion index (C) changed in line with the Total Error Score. The LED + 850 group significantly decreased (mean 1.33 to 1.24; p = 0.01), the LED-only group significantly increased (1.21 to 1.28; p = 0.03), and the control group was unchanged (1.25; p = 0.89). The two intervention groups differed (p = 0.001), and across all sessions C correlated with TE at r = 0.96. The selectivity index showed the errors were not selective (median 1.34, range 1.05 to 1.65) and did not differ between groups. With selectivity low, the confusion angle had no well-defined axis and was not interpreted. Together these indicate a general loss of colour discrimination in this dataset rather than a specific axis effect. The luminaires were indistinguishable to participants as infrared (850nm) is beyond the human visual range [21]. The variability within each of the 3 groups was comparable.

All participants were in similarly designed office environments with white ceilings and light coloured walls. They spent most of their time at their desks in front of PC monitors that were mainly produced by Dell and of a standard form. None were exposed to direct sunlight in the office and all were free to move around. Hence, these results should be seen within the framework of a real-world situation rather than a tightly controlled experimental situation.

## Discussion

We show that standard LED lighting impaired visual colour discrimination in real-world environments. We tested conventional LEDs, typical phosphor-converted warm-white technology (2700K), which minimise blue (450nm), a spectrum expected to be favourable for colour discrimination. But this still lacked the extended infrared range of incandescent lighting that can extend beyond 3000nm.

Blue light (420–450nm) *in vivo* and *in vitro* damages retinal cells and suppresses mitochondrial function, while red wavelengths (650–670nm) enhance mitochondrial activity and improve both vision and systemic metabolism [6,9,22,23]. Near-infrared wavelengths in the range of 800–900nm extend this benefit through deeper tissue penetration, show enhanced cytochrome c oxidase activity in isolated mitochondria, and preserve mitochondrial redox state in rodent retinal photoreceptors *in vivo* and *in vitro* [24–26]. Our results reveal a 33% increase in colour vision error scores with warm white LEDs, suggesting that the absence of longer wavelengths undermines colour vision even when blue emission is minimised. Current blue light hazard limits [27] focus on acute phototoxicity and are orders of magnitude higher than our exposures, implying that chronic low-level effects are not understood. Alternatively, wavelength interactions restricted within the visible spectra may create unpredicted responses, an understudied area, as most research examines single wavelength ranges in isolation.

Recent evidence supports the significance of this spectral imbalance hypothesis. White LED exposure damages human retinal pigment epithelium cell models *in vitro* [28]. Red wavelengths activate repair pathways [28] and counteract damage from shorter wavelengths, both during simultaneous exposure *in vivo* and *in vitro* [29] and as post-exposure treatment *ex vivo* [30]. Our findings translate this evidence from *in vitro* and ex vivo cellular events to human functional outcomes, showing that a spectral imbalance in LEDs produces measurable impairment in colour discrimination. Importantly, supplementation with 850nm in our study both prevented decline and enhanced visual performance in colour vision scores by 23%. This is consistent with evidence that infrared light can stabilise cellular stress responses and enhance mitochondrial function [review 4]. Taken together, our results suggest that colour vision performance depends on access to a spectral range broader than visible light, which explains why even optimally configured visible-only LEDs cause dysfunction.

Mitochondria act as a community across the body. Shifting mitochondrial function locally has systemic consequences. Infrared exposure to the human body improves vision without exposure of the eye [17], consistent with our findings. Infrared exposure to only 5% of the human body surface significantly reduces systemic blood sugars by increasing mitochondrial demand for carbohydrates [9]. Exposure to shorter wavelengths has the opposite effect increasing blood glucose [12]. Additionally, infrared application in Parkinson’s models, which have a mitochondrial basis, is effective when the head is excluded from exposure [31].

Our findings reveal that spectral deficiencies common in real-world lighting environments impair colour vision at a single post-exposure time point. In the context of wider publications mitochondrial dysfunction in cone photoreceptors is likely to be a probable contributor mechanism. Other pathways may contribute. Non-visual opsins (OPN3, OPN4, OPN5) in skin and brain respond to light [32]. Melatonin suppression and circadian disruption are known effects of blue light exposure [33]. While our infrared supplementation at peak 850nm improved colour discrimination, searching for a single-wavelength solution risks oversimplifying the complexity and evolutionary context. Natural sunlight provides full spectrum of visible and invisible wavelengths and engages a variety of chromophores and tissue depths simultaneously in ways that are poorly understood. This is an emerging field of study. Future research should explore restoring spectral diversity through broad-spectrum lighting beyond the visible range, or enhanced daylight access, rather than isolated wavelength supplementation. The rapid and widespread adoption of LED lighting warrants further investigation to understand and address any unintended consequences of spectral restriction on human health, as animal studies *in vivo* reveal LED exposure has widespread diverse physiological consequences [34].

## Supporting information

S1 File Data set used in analysis. (XLSX)

S2 File Spectral irradiance data set. (XLSX)

## References

[1] ASTM International. Standard tables for reference solar spectral irradiances: Direct normal and hemispherical on 37° tilted surface. West Conshohocken, PA: ASTM International. 2003. doi: 10.1520/G0173-03

[2] Zwinkels J. Blackbody and Blackbody Radiation. In: Luo R, editor. Encyclopedia of Color Science and Technology. Springer International Publishing. 2023. p. 110–4. doi: 10.1007/978-3-030-89862-5_370

[3] Klepeis NE, Nelson WC, Ott WR, Robinson JP, Tsang AM, Switzer P, et al. The national human activity pattern survey (NHAPS): A resource for assessing exposure to environmental pollutants. J Expo Anal Environ Epidemiol. 2001;11(3):231–52. doi: 10.1038/sj.jea.7500165 11477521

[4] Hamblin MR. Mechanisms and applications of the anti-inflammatory effects of photobiomodulation. AIMS Biophys. 2017;4(3):337–61. doi: 10.3934/biophy.2017.3.337 28748217 PMC5523874

[5] Begum R, Powner MB, Hudson N, Hogg C, Jeffery G. Treatment with 670 nm light up-regulates cytochrome C oxidase expression and reduces inflammation in an age-related macular degeneration model. PLoS One. 2013;8(2):e57828. doi: 10.1371/journal.pone.0057828 PMC3585189 23469078

[6] Gkotsi D, Begum R, Salt T, Lascaratos G, Hogg C, Chau K-Y, et al. Recharging mitochondrial batteries in old eyes. Near infra-red increases ATP. Exp Eye Res. 2014;122:50–3. doi: 10.1016/j.exer.2014.02.023 24631333

[7] López-Otín C, Blasco MA, Partridge L, Serrano M, Kroemer G. The hallmarks of aging. Cell. 2013;153(6):1194–217. doi: 10.1016/j.cell.2013.05.039 23746838 PMC3836174

[8] Begum R, Calaza K, Kam JH, Salt TE, Hogg C, Jeffery G. Near-infrared light increases ATP, extends lifespan and improves mobility in aged *Drosophila melanogaster*. Biol Lett. 2015;11(3):20150073. doi: 10.1098/rsbl.2015.0073 25788488 PMC4387504

[9] Powner MB, Jeffery G. Light stimulation of mitochondria reduces blood glucose levels. J Biophotonics. 2024;17(5):e202300521. doi: 10.1002/jbio.202300521 38378043

[10] Powner MB, Jeffery G. Systemic glucose levels are modulated by specific wavelengths in the solar light spectrum that shift mitochondrial metabolism. PLoS One. 2022;17(11):e0276937. doi: 10.1371/journal.pone.0276937 36327250 PMC9632789

[11] Kaynezhad P, Fosbury R, Hogg C, Tachtsidis I, Sivaprasad S, Jeffery G. Near infrared spectroscopy reveals instability in retinal mitochondrial metabolism and haemodynamics with blue light exposure at environmental levels. J Biophotonics. 2022;15(4):e202100283. doi: 10.1002/jbio.202100283 35020273

[12] Cheung IN, Zee PC, Shalman D, Malkani RG, Kang J, Reid KJ. Morning and evening blue-enriched light exposure alters metabolic function in normal weight adults. PLoS One. 2016;11(5):e0155601. doi: 10.1371/journal.pone.0155601 27191727 PMC4871543

[13] Kam JH, Hogg C, Fosbury R, Shinhmar H, Jeffery G. Mitochondria are specifically vulnerable to 420nm light in Drosophila which undermines their function and is associated with reduced fly mobility. PLoS One. 2021;16(9):e0257149. doi: 10.1371/journal.pone.0257149 34478469 PMC8415596

[14] Nash TR, Chow ES, Law AD, Fu SD, Fuszara E, Bilska A, et al. Daily blue-light exposure shortens lifespan and causes brain neurodegeneration in *Drosophila*. NPJ Aging Mech Dis. 2019;5:8. doi: 10.1038/s41514-019-0038-6 31636947 PMC6797782

[15] Wong-Riley MTT. Energy metabolism of the visual system. Eye Brain. 2010;2:99–116. doi: 10.2147/EB.S9078 23226947 PMC3515641

[16] Barrett EM, Jeffery G. LED lighting (350-650nm) undermines human visual performance unless supplemented by wider spectra (400-1500nm+) like daylight. Sci Rep. 2026;16(1):3061. doi: 10.1038/s41598-026-35389-6 41577976 PMC12830801

[17] Jeffery G, Fosbury R, Barrett E, Hogg C, Carmona MR, Powner MB. Longer wavelengths in sunlight pass through the human body and have a systemic impact which improves vision. Sci Rep. 2025;15(1):24435. doi: 10.1038/s41598-025-09785-3 40628952 PMC12238558

[18] Farnsworth D. The Farnsworth-Munsell 100-hue and dichotomous tests for color vision. J Opt Soc Am. 1943;33(10):568–78. doi: 10.1364/JOSA.33.000568

[19] Vingrys AJ, King-Smith PE. A quantitative scoring technique for panel tests of color vision. Invest Ophthalmol Vis Sci. 1988;29(1):50–63. 3257208

[20] Hayter AJ. The maximum familywise error rate of Fisher’s least significant difference test. J Am Stat Assoc. 1986;81(396):1000–4. doi: 10.1080/01621459.1986.10478364

[21] Bowmaker JK, Dartnall HJ. Visual pigments of rods and cones in a human retina. J Physiol. 1980;298:501–11. doi: 10.1113/jphysiol.1980.sp013097 7359434 PMC1279132

[22] Kaynezhad P, Tachtsidis I, Jeffery G. Optical monitoring of retinal respiration in real time: 670 nm light increases the redox state of mitochondria. Exp Eye Res. 2016;152:88–93. doi: 10.1016/j.exer.2016.09.006 PMC5105829 27664904

[23] Shinhmar H, Hogg C, Neveu M, Jeffery G. Weeklong improved colour contrast sensitivity after single 670 nm exposures associated with enhanced mitochondrial function. Sci Rep. 2021;11(1):22872. doi: 10.1038/s41598-021-02311-1 PMC8613193 34819619

[24] Amaroli A, Clemente Vargas MR, Pasquale C, Raffetto M, Ravera S. Photobiomodulation on isolated mitochondria at 810 nm: first results on the efficiency of the energy conversion process. Sci Rep. 2024;14(1):11060. doi: 10.1038/s41598-024-61740-w 38744931 PMC11094005

[25] Salehpour F, Mahmoudi J, Kamari F, Sadigh-Eteghad S, Rasta SH, Hamblin MR. Brain photobiomodulation therapy: a narrative review. Mol Neurobiol. 2018;55(8):6601–36. doi: 10.1007/s12035-017-0852-4 29327206 PMC6041198

[26] Gopalakrishnan S, Mehrvar S, Maleki S, Schmitt H, Summerfelt P, Dubis AM, et al. Photobiomodulation preserves mitochondrial redox state and is retinoprotective in a rodent model of retinitis pigmentosa. Sci Rep. 2020;10(1):20382. doi: 10.1038/s41598-020-77290-w 33230161 PMC7684292

[27] International Commission on Non-Ionizing Radiation Protection. Guidelines on limits of exposure to incoherent visible and infrared radiation. Health Phys. 2013;105(1):74–96. doi: 10.1097/HP.0b013e318289a611 35606999

[28] Françon A, Delaunay K, Jaworski T, Lebon C, Picard E, Youale J, et al. Phototoxicity of low doses of light and influence of the spectral composition on human RPE cells. Sci Rep. 2024;14(1):6839. doi: 10.1038/s41598-024-56980-9 38514646 PMC10957882

[29] Núñez-Álvarez C, Osborne NN. Blue light exacerbates and red light counteracts negative insults to retinal ganglion cells in situ and R28 cells in vitro. Neurochem Int. 2019;125:187–96. doi: 10.1016/j.neuint.2019.02.018 30825600

[30] Heinig N, Schumann U, Calzia D, Panfoli I, Ader M, Schmidt MHH, et al. Photobiomodulation mediates neuroprotection against blue light induced retinal photoreceptor degeneration. Int J Mol Sci. 2020;21(7):2370. doi: 10.3390/ijms21072370 32235464 PMC7177783

[31] Johnstone DM, el Massri N, Moro C, Spana S, Wang XS, Torres N, et al. Indirect application of near infrared light induces neuroprotection in a mouse model of parkinsonism - an abscopal neuroprotective effect. Neuroscience. 2014;274:93–101. doi: 10.1016/j.neuroscience.2014.05.023 24857852

[32] Andrabi M, Upton BA, Lang RA, Vemaraju S. An expanding role for nonvisual opsins in extraocular light sensing physiology. Annu Rev Vis Sci. 2023;9:245–67. doi: 10.1146/annurev-vision-100820-094018 37196422

[33] Tähkämö L, Partonen T, Pesonen A-K. Systematic review of light exposure impact on human circadian rhythm. Chronobiol Int. 2019;36(2):151–70. doi: 10.1080/07420528.2018.1527773 30311830

[34] Al-Hussaini H, Al-Onaizi M, Abed BS, Powner MB, Hasan SM, Jeffery G. Impact of short wavelength light exposure on body weight, mobility, anxiety like behaviour and cytokine expression. Sci Rep. 2025;15(1):5927. doi: 10.1038/s41598-025-89081-2 39966413 PMC11836126

